# Boosting Post-Stroke Recovery: The Role of Nutritional Supplementation in Reducing Fatigue—A Single-Blind Randomized Controlled Trial

**DOI:** 10.3390/nu18213506

**Published:** 2026-10-25

**Authors:** Silvia Giovannini, Alessandra Pedico, Anna Maria Malizia, Flavia Angelini, Giovanna Assante Di Panzillo, Claudia Loreti, Nadia Morciano, Giovanni Frisullo, Martina Petrucci, Lorenzo Biscotti, Federico Biscetti, Andrea Flex, Letizia Castelli

**Affiliations:** 1 Department of Geriatrics and Orthopaedics, Università Cattolica del Sacro Cuore, 00168 Rome, Italy; 2 UOSD Riabilitazione Multidimensionale e Tecnologie Integrate, Fondazione Policlinico Universitario A. Gemelli IRCCS, 00168 Rome, Italy; 3 Department of Translational Medicine and Surgery, Università Cattolica del Sacro Cuore, 00168 Rome, Italy; 4 Hospital Health Management, Fondazione Policlinico Universitario A. Gemelli IRCCS, 00168 Rome, Italy; 5 Department of Emergency, Anaesthesiology and Intensive Care Medicine, Fondazione Policlinico Universitario A. Gemelli IRCCS, 00168 Rome, Italy; claudia.loreti@policlinicogemelli.it (C.L.);; 6 UOC Neurologia, Fondazione Policlinico Universitario A. Gemelli IRCCS, 00168 Rome, Italy; 7 Unità Supporto Amministrativo Dipartimenti Universitari, Università Cattolica del Sacro Cuore, 00168 Rome, Italy; 8 UOC Medicina Interna Cardiovascolare, Fondazione Policlinico Universitario A. Gemelli IRCCS, 00168 Rome, Italy; 9 Department of Neurosciences, Università Cattolica del Sacro Cuore, 00168 Rome, Italy; letizia.castelli@unicatt.it

**Keywords:** post-stroke fatigue, neurorehabilitation, nutraceutical supplementation, systemic inflammation, oxidative stress, bioimpedance analysis

## Abstract

Post-stroke fatigue (PSF) remains a debilitating barrier to recovery. Chronic inflammation and oxidative stress often create physiological plateaus that standard rehabilitation alone cannot resolve. This single-blind randomized controlled trial (RCT) evaluated the efficacy of Food for Special Medical Purposes formulated with iron and other vitamins and minerals in Sucrosomial^®^ form, allowing improved absorption, bioavailability, and tolerability, as an adjunct to neurorehabilitation. One hundred and twenty stroke patients were randomized into an experimental group (Sid-G) receiving the specific nutraceutical compound or a control group (CG) receiving standard care. Longitudinal assessments from baseline (T0) through 16 weeks (T3) measured clinical outcomes (including fatigue, motor/cognitive function) and physiological markers, specifically phase angle (PhA) derived from Bioimpedance Analysis, and key inflammatory and oxidative stress biomarkers (IL-6 and MPO). The Sid-G cohort demonstrated superior, sustained improvements in fatigue, motor, and cognitive function (*p* < 0.05). These clinical gains were reflected in the following physiological observations: the time × group interaction for cellular integrity (SPhA) was not significant, though within-group improvements were observed in Sid-G (*p* = 0.009); at the group level, significant time × group interactions were detected for key inflammatory and oxidative stress markers (IL-6, *p* = 0.001; MPO, *p* = 0.026). Correlational analysis revealed a significant association between changes in inflammatory markers and clinical recovery outcomes in Sid-G, a pattern absent in the CG. Adjunctive supplementation may support recovery by helping patients to overcome physiological plateaus. These findings support further evaluation of an integrated, metabolically focused approach to neurorehabilitation.

## 1. Introduction

Post-stroke fatigue (PSF) is one of the most debilitating sequelae of cerebrovascular accidents, affecting a significant portion of survivors and acting as a primary barrier to successful community reintegration [1,2]. Despite its high prevalence and impact on quality of life, the underlying pathophysiology of PSF remains multifaceted, involving a complex interplay between neurobiological changes, metabolic dysfunction, and chronic, low-grade systemic inflammation. While intensive neurorehabilitation is the cornerstone of stroke recovery, many patients reach a “physiological plateau” where further gains in motor and cognitive function are hindered by persistent inflammatory burden and sub-optimal cellular vitality. Recent technological advancements, such as robotic-assisted gait and balance training, have demonstrated significant improvements in patient outcomes by providing high-intensity, task-specific stimuli [3,4,5]. However, even with such targeted mechanical interventions, there is an urgent need to explore integrated therapeutic approaches—such as nutritional supplementation—that address the underlying metabolic substrate of recovery.

Recent scientific focus has increasingly shifted toward the role of nutraceuticals in modulating both physical and cognitive performance. Within this context, the conceptual framework of “medicine–food homology” offers a compelling lens for integrating nutritional interventions into clinical care, recognizing that specialized dietary components can actively modulate metabolic and systemic health. Specifically, addressing issues of nutrient bioavailability and malabsorption in special populations requires advanced delivery strategies, such as pre-digestion and specialized transport mechanisms [6]. Furthermore, the administration of functional lipids and bioactive nutrients has gained traction as a valuable strategy to support neurological recovery, with growing literature documenting how targeted nutrient delivery protects against stroke-induced neurological deficits and associated behavioral symptoms [7,8].

Micronutrients are essential for cellular energy production, mitochondrial function, and the maintenance of structural tissue integrity, all of which directly influence neuromuscular function [9]. Specific compounds have been shown to modulate inflammatory and oxidative pathways [10]. There is growing evidence that iron, magnesium, vitamin D, and various antioxidant compounds may enhance neuromuscular efficiency and alleviate central fatigue, thereby improving both subjective fatigue perception and objective clinical outcomes. The mechanistic hypothesis underpinning this study is that post-stroke patients experiencing persistent fatigue may harbour subclinical deficits in micronutrient availability—driven by malabsorption, elevated inflammatory consumption, or reduced dietary intake during hospitalisation—that impair mitochondrial ATP synthesis, myelin maintenance, and neuromuscular transmission. Restoring micronutrient availability through a multicomponent supplementation strategy may therefore alleviate the metabolic substrate deficit underlying PSF, independently of whether overt deficiency is present at enrolment [11,12]. Moreover, randomized and observational studies consistently indicate that nutritional supplementation can support metabolic recovery and reduce fatigue-related disability in post-stroke and neurological rehabilitation populations [13,14]. Among these, iron—a core component of oxidative metabolism and mitochondrial function—is particularly relevant to meeting the energy demands of post-stroke neural and motor re-learning [15].

Body composition serves as a reliable indicator of a patient’s nutritional and functional status. Parameters such as lean body mass and fluid compartmentalization are closely correlated with metabolic balance and physical performance. The phase angle (PhA), derived from bioimpedance analysis (BIA), is a comprehensive indicator of cellular integrity and fluid distribution, strongly associated with muscle quality and nutritional status [16]. PhA is a recognized prognostic marker for inflammatory burden across multiple neurological disorders [17]. Additionally, alterations in fluid distribution—specifically a reduction in intracellular water and an increase in extracellular water—are markers of catabolic states and impaired muscle function, making them highly relevant in post-stroke management [18]. In patients with chronic and neurological conditions, low PhA levels are associated with malnutrition, sarcopenia, and poorer functional outcomes [19]. Furthermore, recent studies suggest that normalizing these compartments through nutritional intervention can serve as both a prognostic marker and a viable therapeutic target [20].

At the same time, systemic inflammation plays a crucial role in the pathophysiology of fatigue and in post-stroke recovery. Pro-inflammatory cytokines, particularly interleukin-6 (IL-6), are involved in the activation of inflammatory cascades and are associated with disability, sarcopenia, and frailty [21]. More recent evidence indicates that elevated IL-6 levels are associated with worse neurological outcomes, increased fatigue severity, and reduced functional recovery in stroke survivors [22]. IL-6 also exerts a pleiotropic effect in post-stroke neuroinflammation, contributing to both secondary tissue damage and adaptive repair [23]. Conversely, interleukin-10 (IL-10) serves as a critical anti-inflammatory cytokine that promotes the resolution of immune activation [24], and higher IL-10 levels are often associated with improved recovery trajectories and neuroprotection, suggesting its role as a compensatory anti-inflammatory response [25].

Another important biomarker is myeloperoxidase (MPO), a key enzyme in oxidative stress and neuroinflammation that plays a role in the production of reactive oxygen species and is associated with cerebral ischemic damage [26]. Elevated MPO levels have been correlated with infarct size, blood–brain barrier disruption, and worse clinical outcomes after stroke [27], further supporting its role as a marker of oxidative and inflammatory burden. The activation of MPO contributes to the progression of neuronal damage; however, emerging research suggests that nutraceuticals with antioxidant properties may modulate MPO activity and mitigate oxidative burden, thereby promoting neurological recovery [28].

Recent systematic reviews and meta-analyses support the role of targeted nutritional supplementation in improving fatigue, physical performance, and inflammatory profiles, particularly in populations with chronic disease and neurological impairment [13,14,29].

In this context, nutraceuticals may offer therapeutic benefit by modulating systemic inflammation by shifting the balance between pro- and anti-inflammatory cytokines and mitigating oxidative stress. It should be noted that iron deficiency was not an inclusion criterion in the present study; participants were selected based on clinically significant post-stroke fatigue and functional impairment, features consistent with elevated metabolic demand and potential malabsorption risk in the subacute post-stroke phase. Building on the preliminary findings of a pilot study suggesting that SiderAL^®^ Med supplementation significantly improves fatigue, functional performance, and body composition [15], this randomized controlled trial (RCT) was designed to rigorously evaluate the efficacy of SiderAL^®^ Med supplementation on cognitive fatigue, motor performance, and quality of life. By integrating advanced bioimpedance analysis—specifically assessing phase angle and water compartment distribution—and quantifying key biomarkers—including IL-6 and MPO—this study aims to elucidate the pathophysiological mechanisms by which nutraceuticals may serve as a potent, integrated strategy in post-stroke rehabilitation.

## 2. Materials and Methods

This is a single-blind, open-label (with respect to patients and treating therapists), randomized controlled trial. The study design and methodology were developed based on our previously published pilot study [15]. Patients with a history of stroke were randomized into an experimental group (Sid-G) or a control group (CG). The inclusion and exclusion criteria remain consistent with those of the pilot study [15]. Specifically, the inclusion criteria were: (1) age ≥ 18 years, (2) confirmed diagnosis of cerebrovascular accident (stroke), (3) clinical evidence of post-stroke fatigue, and (4) ability to provide informed consent. The exclusion criteria comprised: (1) severe cognitive impairment or aphasia preventing comprehension of study procedures, (2) active severe infections or systemic inflammatory conditions unrelated to stroke, (3) concurrent severe psychiatric disorders, and (4) known hypersensitivity or contraindications to the supplement components. Patients were randomized in a 1:1 ratio to either the experimental or control group using a centralized, computer-generated randomization sequence embedded within a secure, web-based electronic data capture system. Allocation concealment was strictly maintained; the randomization schedule remained inaccessible to all clinical investigators, treating therapists, and outcome assessors until formal baseline enrollment and data entry were completed, thereby preventing selection bias. The manuscript was prepared and revised according to the CONSORT Statement requirements; the participant flow is illustrated in Figure S1 and the completed CONSORT 2025 checklist is provided as Table S1 (see Supplementary Materials). This study was performed in compliance with the national laws and ethical standards established in the 1964 Declaration of Helsinki and its subsequent revisions. The Territorial Ethics Committee (Prot. No. 0031523/22, 29 September 2022) sanctioned the study protocol, and informed consent was secured from each participant before any study procedures commenced (ClinicalTrials.gov ID: NCT05728229, approval date: 4 February 2023).

### 2.1. Measurement

Demographic, anthropometric, clinical, and pharmacological data were collected at baseline (T0). Longitudinal assessments were conducted at baseline (T0), after 4 weeks of hospitalization (T1), at 8 weeks (T2), and at 16 weeks (Follow-up, T3). Given the nature of the interventions, treating therapists and patients were not blinded to group allocation; however, outcome assessors and statistical analysts remained blinded. The success of assessor blinding was not formally evaluated.

With regard to clinical assessment, this included an evaluation of fatigue using the Modified Fatigue Impact Scale (MFIS) [30,31]; an assessment of motor performance using the Motor Index (MI) [32,33], the Berg Balance Scale (BBS) [34], the Short Physical Performance Battery (SPPB) [35], and the Time Up & Go (TUG) test [36]; gait, using the 10-Meter Walking Test (10MWT) and the 6-Minute Walking Test (6MWT) [37], the Hauser Ambulation Index (AI) [38], the Walking Handicap Scale (WHS) [39], and the Functional Ambulation Category (FAC) [40]; cognitive performance, using the Stroop Color Word Test (SCWT) [41], the Symbol Digit Modalities Test (SDMT) [42], and the Trail Making Test (TMT) [43]; independence in activities of daily living (ADL) and quality of life, using the Modified Barthel Index (mBI) [44,45] and the EuroQoL-5D (EQ-5D) [46].

The instrumental assessment included a body composition analysis using bioimpedance analysis (BIA 101 BIVA PRO, Akern S.r.l., Pontassieve, Florence, Italy) with dedicated Bodygram software (Akern S.r.l., Pontassieve, Florence, Italy; www.akern.com), a non-invasive technique that estimates body composition by measuring the resistance (R) and reactance (Xc) of tissues to the passage of a low-intensity electrical current. These parameters allow for the determination of body compartments such as total water, intracellular and extracellular water, lean mass, and fat mass [47,48]. Furthermore, the phase angle (PhA), calculated from Rz and Xc, is considered an indicator of cellular integrity and nutritional status [16]. In stroke rehabilitation, PhA holds high clinical relevance by reflecting cellular health, membrane selectivity, and tissue anabolism, thereby capturing recovery dynamics beyond simple muscle mass changes. Reference normative values stratified by age and sex were considered to benchmark baseline cellular impairment and therapeutic recovery. BIA is widely used in clinical settings to monitor conditions such as malnutrition, sarcopenia, and systemic inflammation [49]. Thanks to its speed and reproducibility, it is a useful tool for functional and prognostic assessment in patients with chronic and neurological conditions.

Key biomarkers were also assessed by measuring levels of IL-6, IL-10, and MPO using commercially available ELISA kits (R&D Systems, Minneapolis, MN, USA): Human IL-6 Quantikine ELISA (cat. D6050), Human IL-10 Quantikine ELISA (cat. D1000B), and Human Myeloperoxidase Quantikine ELISA (cat. DMYE00B), an immunoenzymatic technique widely used for the quantification of protein biomarkers in biological samples, based on specific antigen–antibody interaction. In the case of cytokines and enzymes such as IL-6, IL-10, and MPO, the antigen presented in the sample binds to a specific immobilized antibody, and the subsequent enzymatic reaction generates a colorimetric signal proportional to the concentration of the analyte [50]. ELISA is highly sensitive and specific, making it a widely used and validated method for assessing systemic inflammation [51]. This technique allows for the accurate quantification of pro- and anti-inflammatory cytokines and oxidative enzymes, providing relevant information in clinical and research contexts [52]. It is widely used in studies of cardiovascular and neurological diseases to monitor the inflammatory response and oxidative stress. Serum cytokine and inflammatory markers (IL-6, IL-10, and MPO) were quantified using standard immunoassay procedures according to the manufacturer’s instructions. Values falling below the lower limit of quantification (LLoQ) were handled by substituting them with a value equal to half of the respective LLoQ (LLoQ/2) for statistical evaluation, ensuring an unbiased estimate while maintaining data integrity across longitudinal time points. Serum IL-10 concentrations fell consistently below the lower limit of detection of the assay and were consequently excluded from statistical evaluation.

Analysis of BIA and levels of IL-6, IL-10, and MPO were performed at T0, T2, and T3.

The researchers who performed clinical and instrumental evaluations, and the statistical analysis, were blinded to the randomization of patients into the experimental or control group.

### 2.2. Intervention

As described in the previous study [12], patients deemed eligible were randomized into the Sid-G and the CG. Patients in the Sid-G received one sachet of SiderAL^®^ Med (PharmaNutra S.p.A., Pisa, Italy) per day, taken orally, in addition to their standard pharmacological therapy. SiderAL^®^ Med is a Food for Special Medical Purposes formulated for patients with malabsorption. It contains vitamins B6, B9, B12, D, K, B1, B2, and pantothenic acid, as well as minerals including iron, zinc, magnesium, copper, selenium, calcium, and iodine. Specifically, the daily regimen of 1 sachet provides: elemental iron (as Sucrosomial^®^ Iron) 30 mg, Vitamin C 75 mg, Vitamin E 50 mg, Vitamin PP (Nicotinamide) 5 mg, Vitamin B6 0.7 mg, Vitamin B12 150 mcg, Folate (Vitamin B9) 200 mcg, Pantothenic Acid (Vitamin B5) 5 mg, Vitamin D 75 mcg, Vitamin K 50 mcg, Vitamin A 400 mcg, Vitamin B1 10 mg, Vitamin B2 0.65 mg, Magnesium (UltraMag^®^) 93.75 mg, Zinc (UltraZin^®^) 6 mg, Copper 0.25 mg, Selenium (UltraSel^®^) 30 mcg, Iodine (UltraIod^®^) 37.5 mcg, and Algal Calcium (Aquamin^®^) 200 mg. Most of these components are provided in Sucrosomial^®^ form. Sucrosomial^®^ technology is a patented delivery system in which the active ingredient is conveyed within a phospholipid and sucrester matrix, allowing improved absorption, bioavailability, and tolerability. In this context, the advantage of using SiderAL^®^ Med is to support nutrient delivery in patients with malabsorption, who frequently experience difficulties absorbing micronutrients due to both age-related factors and their inflammatory condition. Compliance was verified through patient-reported logs. Furthermore, safety and tolerability were systematically monitored throughout the study period. Adverse events (AEs), including gastrointestinal symptoms commonly associated with iron supplementation (such as nausea, constipation, or abdominal discomfort), were systematically recorded at each scheduled visit via structured patient interviews and clinical logs. Symptoms were classified as AEs only if they met pre-defined severity thresholds; mild, self-resolving complaints were recorded but not classified as AEs. The intervention was administered during the hospital stay (T0 to T1) and continued for one month post-discharge (T1 to T2). During the subsequent 2 months (T2 to T3), supplementation was discontinued to assess whether effects persisted after withdrawal. The CG received standard pharmacological therapy and rehabilitation alone. All patients underwent identical intensive physical and occupational therapy sessions throughout the T0–T1 period [12], which comprised a structured institutional regimen of conventional motor training, balance exercises, and cognitive re-learning protocols administered consistently according to individual clinical tolerance.

### 2.3. Statistical Analysis

Data were analyzed using SPSS version 25 (IBM Corp., Armonk, NY, USA), with the level of significance set at *p* < 0.05. The allocation sequence was generated using PASS 2023 software (NCSS, LLC, Kaysville, UT, USA) to ensure robust randomization. The primary endpoint of this trial is defined as the change in the Modified Fatigue Impact Scale (MFIS) total score from baseline (T0) to the primary post-intervention endpoint (T2), with all other clinical, functional, bioimpedance, and biomarker assessments designated as secondary or exploratory outcomes. Since this is a study involving a specific subset of patients—for whom the actual efficacy of SiderAL^®^ Med has not yet been investigated in the literature in a large sample of patients—and a formal a priori power calculation targeting a specific minimal clinically important difference (MCID) was not performed prior to enrollment, a retrospective post hoc power analysis confirmed that our completed sample size provides sufficient statistical power (>80%, two-sided α = 0.05) to detect the primary effect size observed in the MFIS. Considering Julious’s practical guidelines [53] for pilot clinical trials and the annual number of patients enrolled, it is estimated that 60 subjects will be included per group, for a total population of 120 subjects.

Baseline variables were summarized using descriptive statistics: continuous data are expressed as mean ± standard deviation (SD) or median and interquartile range (IQR), and categorical data as frequencies and percentages. The Shapiro–Wilk test was applied to assess data distribution. Although several clinical and functional outcomes exhibited non-normal distributions and were thus reported as medians and IQRs, parametric repeated measures (RM) ANOVA was selected for longitudinal and between-group comparisons. Parametric models are robust to non-normality in moderately large samples (n = 60 per group) due to the Central Limit Theorem, and they allow for precise modeling of complex interaction terms across multiple time points (T0 through T3). Within-group longitudinal comparisons across the four time points (T0, T1, T2, and T3) were performed using the RM ANOVA, with the Bonferroni correction for multiple comparisons. To evaluate the efficacy of the intervention between groups, a two-way RM ANOVA was performed between the Sid-G and CG cohorts, and Bonferroni post hoc tests were applied to all significant analyses. This approach allowed for a direct assessment of whether the therapeutic effect observed in the experimental group significantly exceeded that of the control group over the course of the study. To mitigate the risk of Type I errors arising from multiple comparisons across secondary endpoints and longitudinal time points, statistical analyses of secondary outcomes incorporated appropriate corrections where applicable, ensuring rigorous control over the family-wise error rate. The overall significance threshold was set at *p* < 0.05. Bold formatting in the tables indicates values additionally meeting *p* < 0.005 as a marker of the strongest effects; for Bonferroni-corrected post hoc pairwise comparisons, the adjusted threshold was *p* < 0.016 (0.05/3 comparisons), reflecting the three sequential treatment phases of the study design (T0-T1 hospitalization, T1-T2 post-discharge, T2-T3 follow-up); the T0-T3 contrast was included as a descriptive overall change estimate and was not part of the corrected family of comparisons. Furthermore, to account for potential confounding factors, any multivariable models evaluating the relationship between inflammatory reductions and functional recovery were adjusted for key clinical covariates, including age, sex, baseline stroke severity, and major comorbidities. To address the baseline imbalance in SPhA and to provide between-group estimates adjusted for pre-treatment differences, ANCOVA analyses were additionally performed for the primary and key secondary endpoints, with baseline scores as covariates. Monotone relationships between biological biomarkers and longitudinal or cross-sectional clinical variables were assessed using Spearman’s rank correlation coefficient (ρ), given the non-normal distribution of the variables. Statistical significance for bivariate unadjusted correlation analyses was set at *p* < 0.05, considering the sample size of 60 subjects per study group (N = 60). All statistical tests employed were two-tailed.

## 3. Results

### 3.1. Participant Characteristics and Study Flow

A total of 120 patients who met the inclusion criteria were enrolled and randomized into the experimental (Sid-G) and control (CG) groups. The baseline characteristics of the study cohort closely reflect the broader population of subacute stroke survivors routinely managed in specialized rehabilitation settings. By specifically capturing patients presenting with post-stroke fatigue and functional impairment while excluding those with severe confounding cognitive deficits or unrelated acute inflammatory conditions, the cohort ensures a representative cross-section of real-world clinical practice in this patient demographic. The demographic, clinical, and functional characteristics of the 120 participants are presented in Table 1. At baseline, the experimental (Sid-G) and control (CG) groups were well-matched in terms of age, body mass index (BMI), and sex distribution (*p* > 0.05). Baseline assessments of fatigue (MFIS subscales and total score), functional motor performance, and cognitive status revealed no statistically significant differences between the two study arms. Furthermore, biomarkers (IL-6 and MPO) and most body composition parameters were comparable at study entry. The detectable IL-10 levels were below 0.78 pg/L and were therefore not detectable during analysis. For this reason, they were not reported in Table 1 or considered in the subsequent data analysis. Since the PhA is strongly influenced by age and sex, the analysis was conducted primarily using the Standardized Phase Angle (SPhA), which expresses the individual value as a deviation from the reference values of the healthy population. This standardization reduces variability associated with demographic characteristics and allows for a more accurate comparison between individuals and groups, thereby improving the clinical interpretability of the observed differences [54,55]. While a significant difference was noted in the SPhA (*p* = 0.007), all other physiological and clinical markers confirmed that the study cohorts were balanced prior to the implementation of the nutritional intervention.

### 3.2. Longitudinal Recovery Trajectories

The longitudinal assessment revealed distinct recovery trajectories between the two study arms. Both groups demonstrated significant improvements in clinical and functional measures over the 16-week study period (*p* < 0.001 for time effects). However, the Sid-G exhibited more sustained recovery patterns, particularly in cognitive fatigue (MFIS Cog) and gait-related performance (10MWT, 6MWT), with significant improvements maintained into the follow-up period (T2-T3) (Table 2).

The physiological and analytical data in Table 3 highlight the specific impact of the SiderAL^®^ Med supplementation. The Sid-G showed statistically significant longitudinal changes in markers of cellular integrity and metabolic status, including the SPhA (*p* = 0.009) and Skeletal Muscle Mass (SMM, *p* = 0.011) within the Sid-G. It should be noted that while these within-group changes were statistically significant, the corresponding time × group interaction for SPhA was not (*p* = 0.590, Table 5); changes in SPhA should therefore not be attributed specifically to the supplementation when compared with the control group. Furthermore, the nutraceutical intervention was associated with significant longitudinal changes in key biomarkers of oxidative stress, with favorable shifts in IL-6 (*p* = 0.049) and MPO (*p* < 0.001) levels. In contrast, the CG failed to achieve statistical significance in these physiological and biochemical domains, despite participating in the same intensive rehabilitation protocol.

ANCOVA of the primary endpoint confirmed a significant between-group difference in MFIS Total at T2 after adjustment for baseline (adjusted mean difference Sid-G vs. CG: –6.2 points, 95% CI [–11.2, –1.2], *p* = 0.015). ANCOVA also confirmed significant group differences for MFIS Physical (*p* < 0.001), MFIS Cognitive (*p* < 0.001), BBS (*p* < 0.001), and SPPB (*p* < 0.001) at T2. The two-way repeated measures ANOVA confirmed a significant interaction effect (time × group) across primary and secondary endpoints, indicating that the recovery trajectories were significantly influenced by the addition of the nutraceutical intervention. Sid-G demonstrated superior progress in fatigue management (MFIS Total, *p* = 0.040) and physical performance (BBS, *p* < 0.001; SPPB, *p* = 0.007; 6MWT, *p* < 0.001) as shown in Table 4.

Figure 1 graphically illustrates the more pronounced and rapid reduction in fatigue scores (MFIS) and an increase in physical performance (6MWT, BBS, and SPPB) in the Sid-G compared to the CG.

These clinical gains were mirrored by significant physiological changes; the interaction analysis revealed a differential effect on oxidative markers and body composition parameters in the Sid-G compared with the CG, with significant time × group interactions for IL-6 (*p* = 0.001), MPO (*p* = 0.026), SMM (*p* = 0.007), and Hydration (*p* < 0.001) (Table 5).

**Table 5:** Two-way repeated measures ANOVA and post hoc analysis for BIA, Il-6 and MPO.

Sid-G vs. CG					
			Post Hoc Test		
	*p* Time	*p* Time × Group	T0-T2	T2-T3	T0-T3 ^†^
Hydration	0.151	**<0.001**	**<0.001**	**<0.001**	0.125
SMI	0.242	0.844	-	-	-
SMM	**0.005**	**0.007**	0.050	0.153	0.886
ASMM	**0.009**	0.128	-	-	-
ASMMI	**0.003**	0.067	-	-	-
SPhA	0.454	0.590	-	-	-
IL-6	**0.024**	0.001	**0.028**	0.050	**0.013**
MPO	**0.018**	**0.026**	**0.022**	0.367	**0.014**

The significant values for *p* < 0.005 are in bold. The significant values from the post hoc test (*p* < 0.016, based on three planned comparisons: T0-T1, T1-T2, T2-T3) are in bold. ^†^ T0-T3 values are exploratory overall change estimates, not included in the Bonferroni-corrected family of comparisons.

Regarding fluid homeostasis, the Sid-G demonstrated a significant modulation in hydration levels compared to the CG, with a marked reduction between T0 and T2 (*p* < 0.001), and an increase between T2 and T3 (*p* < 0.001).

At the same time, a statistically significant change in key biomarkers in the Sid-G compared to the CG was observed. Based on unadjusted exploratory comparisons spanning the full follow-up (T0-T3), IL-6 levels showed a decrease between T0 and T3 (*p* = 0.013); similarly, a decrease in MPO levels was observed between T0 and T3 (*p* = 0.014).

These results are shown graphically in Figure 2.

### 3.3. Correlational Analysis: Clinical-Biological Interplay

To elucidate the relationship between systemic biomarkers, cellular integrity, and clinical recovery, Spearman’s rank correlation analysis was performed (Table 6 and Table 7).

In the Sid-G (Table 6), significant associations between biochemical markers and clinical outcomes were identified. While the correlation between changes in IL-6 (ΔIL-6) and fluid homeostasis (ΔHydration) was statistically significant (*p* = 0.015), the effect size was small (rho = −0.312), suggesting a minor homeostatic trend. More importantly, both Physical (rho = 0.528, *p* < 0.001) and Cognitive (rho = 0.456, *p* < 0.001) fatigue at T3 were positively correlated with IL-6 levels.

The strong correlation between IL-6 and MPO at T3 (rho = 0.888, *p* < 0.001) demonstrates a strong statistical correlation between oxidative and inflammatory markers, suggesting a shared inflammatory-oxidative pathway profile during follow-up.

In contrast, the Control Group (Table 7) displayed a markedly different correlational profile. While the relationship between IL-6 and MPO remained consistent at follow-up (rho = 0.697, *p* < 0.001), the clinical-biological links observed in the Sid-G were absent in the CG. Specifically, the associations between ΔIL-6 and ΔMFIS failed to reach statistical significance (*p* > 0.05). Furthermore, no significant relationship was observed between T3 IL-6 levels and fatigue scores (Physical, *p* = 0.096; Cognitive, *p* = 0.114).

Regarding safety outcomes, the intervention was well tolerated overall. No formal adverse events related to the supplementation were recorded in either group throughout the study period. Two participants in the Sid-G reported mild, transient gastrointestinal discomfort (nausea) that resolved spontaneously and did not meet the pre-defined criteria for classification as an adverse event; no such complaints were reported in the CG. No participant discontinued the intervention due to tolerability concerns, confirming an excellent safety profile for SiderAL^®^ Med at the administered dose.

## 4. Discussion

Post-stroke fatigue remains one of the most debilitating yet poorly managed sequelae of cerebrovascular accidents, significantly impeding functional recovery and quality of life [56]. Despite the recognized role of oxidative stress in the pathophysiology of PSF [57], clinical management has largely remained confined to standardized rehabilitation protocols, often leaving the underlying metabolic and inflammatory substrates of the disease unaddressed. The present study aimed to bridge this gap by evaluating the efficacy of SiderAL^®^ Med—a multicomponent nutraceutical—as an adjunct to intensive neurorehabilitation, hypothesizing that targeted metabolic support could fundamentally alter the recovery trajectory of stroke survivors.

The findings of this RCT suggest that adjunctive supplementation with SiderAL^®^ Med significantly enhances recovery trajectories in patients undergoing post-stroke neurorehabilitation. These results substantiate the proof-of-concept established in the previous pilot study [15], which first identified the potential of this intervention to improve fatigue and body composition in a smaller cohort.

By integrating recent evidence on nutritional immunomodulation, this study confirms that these improvements are driven by a targeted metabolic shift. Specifically, the changes in key biomarkers (IL-6 and MPO) related to inflammation and oxidation suggest a possible mitigation of the systemic inflammatory status, common in post-stroke recovery [10]. This is critical, as recent research indicates that chronic inflammatory states actively impair neuroplasticity and recovery processes within the central nervous system [58]. Furthermore, the observed improvements in functional motor outcomes and autonomy underscore the importance of nutritional support in sustaining the metabolic demands of intensive physiotherapy, aligning with emerging clinical paradigms that prioritize metabolic optimization alongside traditional rehabilitation [59]. By validating these findings in a larger, more diverse population and elucidating the underlying physiological mechanisms through a detailed analysis of key biomarkers, this study suggests that the intervention may act as a modulator of the metabolic substrate required for functional restoration. It should be noted that changes in SPhA within the Sid-G, while statistically significant at the within-group level (*p* = 0.009), did not reach significance in the time × group interaction analysis (*p* = 0.590); SPhA changes should therefore be interpreted as reflecting shared rehabilitation effects rather than a supplement-specific effect.

These findings align with and extend existing literature suggesting that post-stroke recovery is frequently impeded by chronic, low-grade systemic inflammation and elevated oxidative stress, which correlate strongly with persistent fatigue and diminished functional autonomy [60,61,62]. The significant interaction effects observed across our primary clinical endpoints—specifically the MFIS and the 6MWT—suggest that the efficacy noted in the pilot trial was maintained in this large cohort, while providing a critical layer of biological evidence. These findings were further supported by ANCOVA analyses adjusting for baseline differences, which confirmed a significant between-group difference in MFIS Total at T2 (adjusted mean difference: −6.2 points, 95% CI [−11.2, −1.2], *p* = 0.015), reinforcing the robustness of the primary endpoint result. While recent innovations such as robotic-assisted training have revolutionized physical therapy by improving balance and gait through high-intensity, task-specific feedback [3,63,64], these mechanical approaches often encounter a ‘physiological plateau’ when the patient’s metabolic substrate is deficient. These findings suggest that concurrent nutritional supplementation may act in parallel to these technologies. At the cellular level, we hypothesize that targeted metabolic support helps overcome these plateaus through three interconnected pathways: first, by optimizing mitochondrial function and cellular respiration—relieving iron-dependent bottlenecks in ATP synthesis that compound post-stroke fatigue; second, by attenuating systemic inflammation and oxidative injury, thereby mitigating MPO-mediated blood–brain barrier dysfunction and secondary damage; and third, by supplying the essential cofactors (such as B-vitamins, minerals, and Sucrosomial^®^ iron) required to support myelin synthesis, membrane stabilization, and neuroplastic adaptation during intensive motor rehabilitation. The trend of IL-6 and MPO observed in the experimental group suggests that SiderAL^®^ Med supports the body’s natural compensatory mechanisms during the critical post-acute recovery phase. This finding is supported by research linking these specific cytokines to impaired long-term functional and cognitive outcomes [65] and given that MPO is a key driver of secondary brain injury and blood–brain barrier dysfunction following stroke, and its inhibition is increasingly recognized as a viable strategy to improve long-term neurological prognosis [66]. Improvements in SPhA, a validated proxy for cellular health and membrane integrity [67], and SMM suggest that the intervention contributes to enhanced cellular vitality, which is essential for sustaining the metabolic demands of neural and motor re-learning post-stroke [68]. The nuanced trajectory of IL-6 and MPO, characterized by a dynamic pattern with the most pronounced changes between T0 and T2 followed by partial recovery after supplementation discontinuation at T2, warrants careful interpretation. Rather than reflecting a simple continuous reduction, this time-window-dependent pattern may suggest a transient modulatory effect that attenuates after withdrawal; future studies should examine whether continuous or longer supplementation sustains these biomarker changes. Future clinical studies could explore targeted nutritional screening (incorporating inflammatory profiles and bioimpedance phase angle assessments alongside motor scores) to identify metabolic vulnerabilities, and investigate the integration of supplementation as a potential adjunct to intensive rehabilitation, with structured administration timelines to evaluate individual sustainability [69], potentially preventing the transition from adaptive repair to chronic, deleterious inflammation [68,69].

A noteworthy finding of this study is the significant association revealed by our correlational analysis. The Sid-G group demonstrated a consistent link between changes in inflammatory biomarkers and clinical fatigue alleviation—a pattern absent in the control arm. This suggests that without targeted nutritional support, patients may reach a physiological plateau that standard rehabilitation alone cannot overcome [70,71]. These findings position nutritional intervention as a critical, underutilized pillar of neurorehabilitation [72]. The present findings are consistent with a “metabolic ceiling” model of recovery: if the biological “floor” of a patient’s inflammatory and metabolic state is too unfavorable, their capacity for functional motor recovery may be constrained. Should this hypothesis be confirmed in future placebo-controlled trials, addressing this biological floor through nutritional supplementation could enhance the efficacy of rehabilitation protocols. However, we must exercise caution regarding causality; while the observed correlations between fatigue reduction and the modulation of IL-6 and MPO highlight a compelling link, these findings reflect associations rather than definitive proof that the supplement mediated fatigue alleviation exclusively through these inflammatory and oxidative pathways.

These outcomes align closely with contemporary nutritional and neurological research emphasizing the therapeutic value of specialized dietary components and functional lipids in ameliorating post-stroke sequelae and modulating neuroinflammation [7,8], while supporting the broader clinical application of medicine–food homology frameworks. Moreover, the observed modulation of myeloperoxidase (MPO) and systemic inflammatory markers resonates with recent mechanistic findings demonstrating that targeted interventions can effectively suppress oxidative stress and related pathways (such as ferroptosis and associated inflammatory cascades) during cerebral ischemia and recovery [73], reinforcing the biological plausibility of our findings.

While these results are promising, this study has limitations that warrant consideration. First, the absence of a placebo control group is the single most important limitation for the interpretation of subjective outcomes. The single-blind design—wherein outcome assessors were blinded but treating therapists and patients were not—combined with the lack of a parallel inactive matched sachet, introduces a meaningful risk of performance, expectancy, and response bias, particularly for subjective outcome measures such as the MFIS, where participant awareness of receiving an active nutritional supplement could amplify reported symptomatic improvements via placebo mechanisms; future confirmatory trials should incorporate double-blind, placebo-controlled designs to fully isolate these effects. Second, although the sample size of 60 patients per group was initially estimated based on institutional recruitment capacity and feasibility rules rather than an a priori power calculation, subsequent post hoc evaluations confirm that this sample size provided adequate statistical power (>80% at α = 0.05) to detect the observed effects on primary outcomes; nonetheless, the absence of an initial formal power estimation represents a methodological limitation that will be addressed in future multi-center RCTs. Third, while the follow-up period of 16 weeks captured the immediate post-discontinuation window, future investigations must extend observation periods to definitively establish long-term sustainability. Fourth, while the current findings demonstrate associations for this specific Sucrosomial^®^ formulation, validation across alternate nutritional strategies is warranted. Furthermore, serum markers of iron status (ferritin, haemoglobin, transferrin saturation) were not measured; their absence limits the ability to attribute the observed effects to iron specifically or to confirm that iron deficiency was corrected by the intervention. Fifth, mechanistic insights derived from systemic biomarkers should be complemented by advanced neuroimaging in subsequent studies to directly map central nervous system microenvironment changes. Finally, multi-center trials involving diverse clinical settings and broader patient demographics are necessary to confirm generalizability.

Future research should focus on long-term longitudinal monitoring to determine if these metabolic benefits translate into reduced risks of recurrence and on exploring whether specific patient phenotypes derive the greatest benefit from this adjunctive approach.

## 5. Conclusions

The present RCT demonstrates that the targeted nutritional supplementation of SiderAL^®^ Med, when integrated into a standard intensive neurorehabilitation protocol, is associated with improvements in post-stroke fatigue, body composition parameters, and specific systemic inflammatory/oxidative biomarkers. By modulating energy metabolism and key biomarkers, this intervention is associated with a more robust and resilient recovery trajectory than rehabilitation alone. The clinical benefits—specifically the significant reduction in fatigue and improvement in motor and functional capacity—are consistent with a favorable shift in the metabolic and biological substrate of the patient, though the precise mechanisms remain to be established in future controlled studies. The observed association between biomarker modulation and clinical progress suggests that mitigating the oxidative and inflammatory “burden” may contribute to overcoming the physiological plateaus often encountered in the post-acute phase.

From a clinical perspective, these findings suggest potential directions for future clinical investigation. Future clinical studies could explore targeted nutritional screening (incorporating inflammatory profiles and bioimpedance phase angle assessments alongside motor scores) to identify metabolic vulnerabilities and investigate the integration of supplementation as a potential adjunct to intensive or robotic-assisted physical therapies, with structured administration timelines and planned discontinuation windows to evaluate individual sustainability.

Regarding real-world implementation, SiderAL^®^ Med—as a Food for Special Medical Purposes utilizing Sucrosomial^®^ technology—offers high gastrointestinal tolerability, excellent absorption, and a convenient once-daily oral administration that avoids the severe adverse effects and compliance hurdles of conventional iron therapies. Consequently, it introduces minimal logistical burden or infrastructure costs compared to advanced technological interventions. However, successful integration into broader healthcare systems will rely on clinician education to match nutritional protocols with specific patient metabolic phenotypes, alongside institutional cost-effectiveness evaluations. In conclusion, this RCT provides preliminary evidence supporting further investigation of the multicomponent nutritional supplementation (SiderAL^®^ Med) as an adjunctive strategy in neurorehabilitation programs. If confirmed in future placebo-controlled trials, elevating this biological floor through nutritional supplementation could enable rehabilitation protocols to achieve greater therapeutic efficacy. These results offer a strong foundation for redefining comprehensive post-stroke management by addressing the critical metabolic and systemic barriers to recovery.

## Figures and Tables

**Figure 1:**
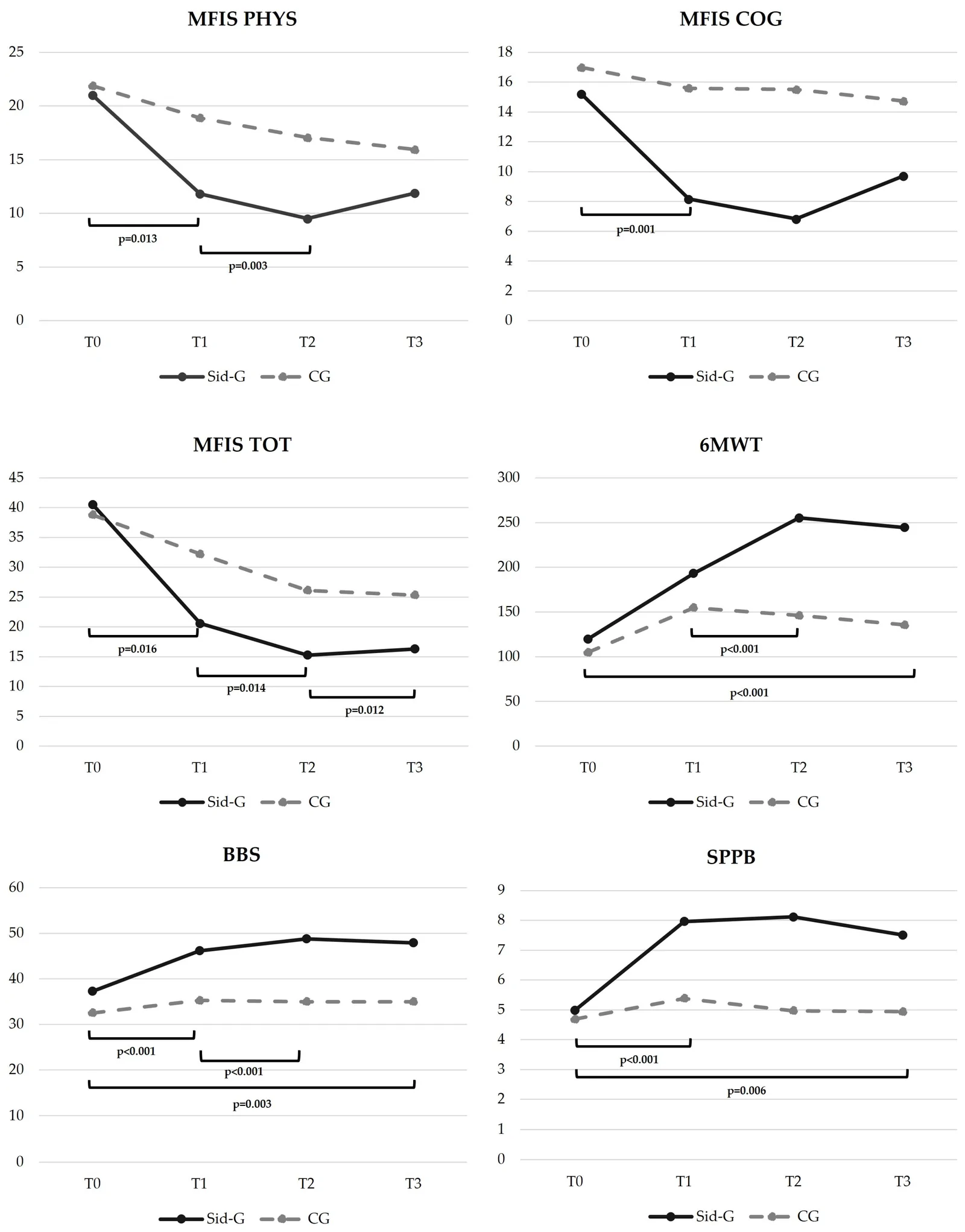
**Longitudinal trends in clinical assessments of the Sid-G and CG cohorts.** The figure illustrates the mean progression of patients in the Sid-G (experimental group, solid black line) and the CG (control group, dashed gray line) across time points T0, T1, T2, and T3.

**Figure 2:**
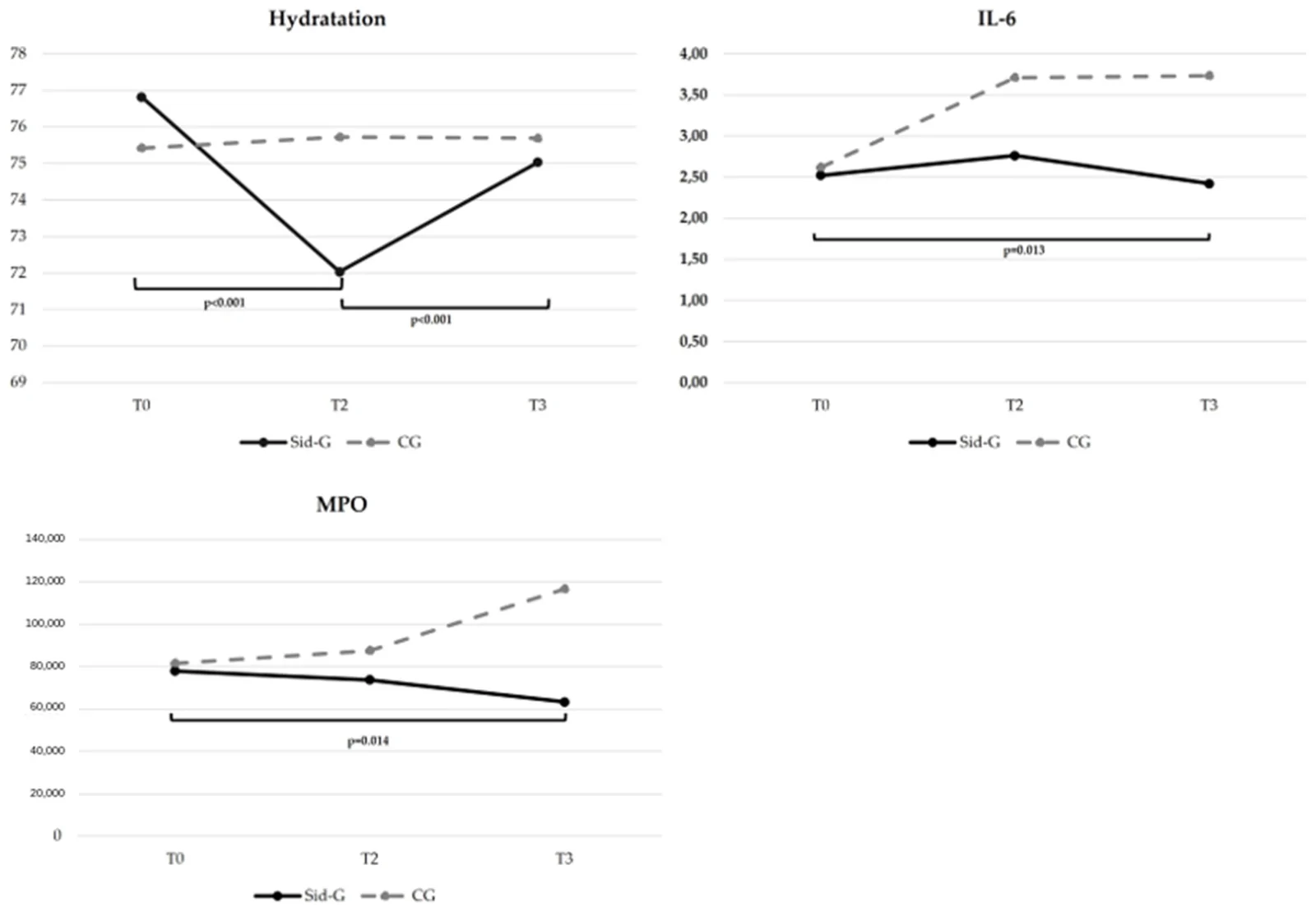
**Temporal evolution of metabolic and oxidative stress biomarkers.** Trends in systemic biomarkers and body composition indicators are shown for the Sid-G (solid black line) and CG (dashed gray line).

**Table 1:** Clinical and demographic characteristics of the population at baseline.

		Sid-G (n = 60)	CG (n = 60)	*p* Value
Gender, W vs. M	n	27 vs. 33	29 vs. 31	0.745
Age, years	mean ± SD	70.4 ± 12.54	72.60 ± 12.93	0.465
BMI	mean ± SD	26.60 ± 3.762	26.52 ± 4.48	0.834
MFIS Phy	median (IQR)	21 (17.5–25)	22.50 (20–24)	0.407
MFIS Cog	median (IQR)	14 (11–19)	18.50 (12.25–21)	0.061
MFIS Psy	median (IQR)	4 (3–6)	4 (3–6)	0.443
MFIS Tot	median (IQR)	40.50 (32.75–54.25)	44 (30.25–51.25)	0.927
EQ-5D VAS	median (IQR)	60 (50–65)	55 (45–65)	0.540
mBI	median (IQR)	59 (38–73.50)	38 (33.75–65.0)	0.069
MI Affected side	median (IQR)	58 (48–70)	58 (58–64)	0.862
BBS	median (IQR)	37 (32.50–42.50)	35 (26–39)	0.060
SPPB	median (IQR)	4 (3–6.50)	4 (3–6)	0.878
AI	median (IQR)	3 (3–4)	4 (3–5)	0.070
FAC	median (IQR)	3 (2–4)	3 (2–3)	0.061
WHS	median (IQR)	4 (3–5)	4 (2–4)	0.671
TUG, seconds	median (IQR)	20.25 (12.77–31.73)	26.31 (18.66–46)	0.072
10MWT, seconds	median (IQR)	30.04 (20.18–36.30)	28.87 (26.66–74.41)	0.340
6MWT, meters	median (IQR)	100 (70–130)	100 (80–145)	0.933
SDMT	median (IQR)	13 (8.50–20)	12 (9–20.25)	0.934
TMT B-A	median (IQR)	50.98 (19.19–76.31)	32 (15.92–60.83)	0.132
SCWT, seconds	median (IQR)	66.31 (36.75–89.29)	64.30 (35.17–99)	0.070
SCWT, errors	median (IQR)	4 (1–5.50)	5 (3–7)	0.088
Rz	median (IQR)	506.80 (454.93–536.38)	526.65 (452.91–559.77)	0.209
Xc	median (IQR)	39.20 (34.42–42.80)	36.25 (33.30–50.15)	0.552
PhA	median (IQR)	4.30 (4–4.70)	4.5 (3.40–5.5)	0.286
Hydration	median (IQR)	75.50 (73.80–77.23)	74.80 (73.50–78.30)	0.940
SMI	median (IQR)	8.95 (7.40–9.70)	8.40 (7–8.80)	0.097
SMM	median (IQR)	24.75 (19.72–28.80)	21.55 (18.60–26.85)	0.403
ASMM	median (IQR)	20.20 (17.15–22.43)	18.40 (16.55–20.80)	0.459
ASMMI	median (IQR)	6.95 (6.32–7.40)	6.85 (6–7.70)	0.795
SPhA	median (IQR)	−1.22 (−1.66–0.66)	−1.42 (−1.82–0.98)	**0.007**
IL-6	median (IQR)	2.20 (1.36–3.21)	2.61 (1.82–3.64)	0.113
MPO (pmol/L)	median (IQR)	69,290 (59,482.50–87,890.00)	73,555 (50,577.50–132,000.00)	0.407

BMI: Body Mass Index; MFIS: Modified Fatigue Impact Scale; Phy: Physical; COG: Cognitive; Psy: Psychosocial; MI: Motricity Index; BBS: Berg Balance Scale; SPPB: Short Physical Performance Battery; AI: Ambulation Index; FAC: Functional Ambulation Category; WHS: Walking Handicap Scale; TUG: Timed Up&Go Test; 10MWT: 10-Metre Walking Test; 6MWT: 6-Minute Walking Test; SDMT: Symbol Digit Modalities Test; TMT: Trial Making Test; SCWT; Stroop Colour Word Test; EQ-5D: EuroQoL-5D; mBI: modified Barthel Index; Rz: resistance; Xc: reactance; PhA: phase angle; SMI: Skeletal Muscle Index; SMM: Skeletal Muscle Mass; ASMM: Appendicular Skeletal Muscle Mass; ASMMI: Appendicular Skeletal Muscle Mass Index; SPhA: Standardized Phase Angle; IL-6: Interleukin-6; MPO: Myeloperoxidase. Statistically significant *p*-values of *p* < 0.005 are in bold.

**Table 2:** RM-ANOVA and post hoc analysis between the assessment times in the two groups for clinical assessments.

	Sid-G					CG				
	*p* Time	Post Hoc Test				*p* Time	Post Hoc Test			
		T0-T1	T1-T2	T2-T3	T0-T3 ^†^		T0-T1	T1-T2	T2-T3	T0-T3 ^†^
MFIS Phy	**<0.001**	**<0.001**	**<0.001**	0.022	**<0.001**	**<0.001**	**<0.001**	**<0.001**	0.039	**<0.001**
MFIS Cog	**<0.001**	**<0.001**	0.005	0.055	**<0.001**	**<0.001**	**<0.001**	0.989	0.031	**<0.001**
MFIS Psy	**<0.001**	**<0.001**	0.269	0.099	**<0.001**	**<0.001**	**<0.001**	0.032	0.135	**<0.001**
MFIS Tot	**<0.001**	**<0.001**	**<0.001**	0.450	**<0.001**	**<0.001**	**<0.001**	**0.001**	**0.005**	**<0.001**
EQ-5D VAS	**<0.001**	**<0.001**	0.077	**0.004**	**<0.001**	**<0.001**	**<0.001**	0.025	**0.008**	**<0.001**
mBI	**<0.001**	**<0.001**	**<0.001**	0.504	**<0.001**	**<0.001**	**<0.001**	0.411	0.057	**<0.001**
MI Affected side	**<0.001**	**<0.001**	**0.001**	0.036	**<0.001**	**<0.001**	**<0.001**	0.174	0.346	**<0.001**
BBS	**<0.001**	**<0.001**	**<0.001**	0.919	0.077	**<0.001**	**<0.001**	**<0.001**	0.286	**0.001**
SPPB	**<0.001**	**<0.001**	0.721	0.369	0.001	**<0.001**	**<0.001**	**<0.001**	0.484	**0.005**
AI	**<0.001**	**<0.001**	**<0.001**	**0.014**	**<0.001**	**<0.001**	**<0.001**	0.031	0.413	**<0.001**
FAC	**<0.001**	**<0.001**	0.065	0.168	**<0.001**	0.648	-	-	-	-
WHS	**<0.001**	**0.002**	**0.007**	0.314	**<0.001**	**<0.001**	**<0.001**	0.484	0.745	**<0.001**
TUG	**<0.001**	**<0.001**	0.797	**<0.001**	**<0.001**	**0.009**	**<0.001**	**<0.001**	**0.014**	**0.015**
10MWT	**<0.001**	**<0.001**	0.026	**<0.001**	**0.001**	**0.012**	**<0.001**	**<0.001**	**<0.001**	**0.008**
6MWT	**<0.001**	**<0.001**	**<0.001**	**<0.001**	**<0.001**	**<0.001**	**<0.001**	**0.002**	**<0.001**	**<0.001**
SDMT	**<0.001**	**<0.001**	0.412	0.218	**<0.001**	**<0.001**	**<0.001**	**<0.001**	0.041	**<0.001**
TMT B-A	**0.014**	0.589	0.541	0.030	**0.006**	**0.016**	0.018	0.319	0.019	0.021
SCWT, seconds	**<0.001**	**0.004**	0.087	0.371	**0.005**	**0.001**	**<0.001**	**<0.001**	0.406	**0.001**
SCWT, errors	**<0.001**	**<0.001**	0.691	0.514	**<0.001**	**0.001**	**<0.001**	**0.005**	0.830	**0.001**

The significant values for *p* < 0.005 are in bold. The significant values from the post hoc test (*p* < 0.016, based on three planned comparisons: T0-T1, T1-T2, T2-T3) are in bold. ^†^ T0-T3 values are exploratory overall change estimates, not included in the Bonferroni-corrected family of comparisons.

**Table 3:** RM-ANOVA and post hoc analysis between the assessment times in the two groups for BIA, Il-6 and MPO.

	Sid-G				CG			
	*p* Time	Post Hoc Test			*p* Time	Post Hoc Test		
		T0-T2	T2-T3	T0-T3 ^†^		T0-T2	T2-T3	T0-T3 ^†^
Hydration	**0.046**	**<0.001**	0.312	**<0.001**	0.842	-	-	-
SMI	0.161	-	-	-	0.489	-	-	-
SMM	**0.011**	0.688	0.392	0.415	0.879	-	-	-
ASMM	**0.010**	0.166	0.898	0.342	0.317	-	-	-
ASMMI	**0.002**	0.326	**0.014**	**0.002**	0.418	-	-	-
SPhA	**0.009**	0.640	**<0.001**	0.028	0.962	-	-	-
IL-6	**0.049**	0.560	0.012	0.288	0.633	-	-	-
MPO	**<0.001**	**<0.001**	0.139	**0.022**	0.535	-	-	-

The significant values for *p* < 0.005 are in bold. The significant values from the post hoc test (*p* < 0.016, based on three planned comparisons: T0-T1, T1-T2, T2-T3) are in bold. ^†^ T0-T3 values are exploratory overall change estimates, not included in the Bonferroni-corrected family of comparisons. Dashes (“-”) indicate that post hoc pairwise comparisons were omitted due to a lack of statistical significance in the overall RM-ANOVA time effect (*p* > 0.05).

**Table 4:** Two-way repeated measures ANOVA and post hoc analysis for clinical assessments.

	Sid-G vs. CG					
				Post Hoc Test		
	*p* Time	*p* Time × Group	T0-T1	T1-T2	T2-T3	T0-T3 ^†^
MFIS Phy	**<0.001**	**0.008**	**0.013**	**0.003**	0.098	0.128
MFIS Cog	**<0.001**	**0.037**	0.090	**0.001**	0.044	0.798
MFIS Psy	**<0.001**	0.162	-	-	-	-
MFIS Tot	**<0.001**	**0.040**	**0.016**	**0.014**	**0.012**	0.083
EQ-5D VAS	**<0.001**	0.698	-	-	-	-
mBI	**<0.001**	0.064	-	-	-	-
MI Affected side	**<0.001**	0.273	-	-	-	-
BBS	**<0.001**	**<0.001**	**<0.001**	**<0.001**	0.245	**0.003**
SPPB	**<0.001**	**0.007**	**<0.001**	0.151	0.058	**0.006**
AI	**<0.001**	**<0.001**	**0.006**	0.197	0.424	**0.015**
FAC	**<0.001**	**<0.001**	**<0.001**	0.977	**<0.001**	**0.008**
WHS	**<0.001**	0.110	-	-	-	-
TUG	**<0.001**	**0.010**	0.852	0.138	0.481	0.355
10MWT	**<0.001**	0.401	-	-	-	-
6MWT	**<0.001**	**<0.001**	0.082	**<0.001**	0.632	**<0.001**
SDMT	**<0.001**	**0.005**	0.332	0.669	0.642	0.453
TMT B-A	**<0.001**	**0.008**	**0.004**	0.282	0.019	0.485
SCWT, seconds	**<0.001**	0.923	-	-	-	-
SCWT, errors	**<0.001**	0.070	-	-	-	-

The significant values for *p* < 0.005 are in bold. The significant values from the post hoc test (*p* < 0.016, based on three planned comparisons: T0-T1, T1-T2, T2-T3) are in bold. ^†^ T0-T3 values are exploratory overall change estimates, not included in the Bonferroni-corrected family of comparisons.

**Table 6:** Spearman’s correlation analysis between clinical and biological variables in Sid-G.

	Spearman’s ρ	*p* Value
ΔIL-6 (T0-T3) vs. Δ Hydration (T0-T3)	−0.312	**0.015**
ΔIL-6 (T0-T3) vs. Δ SPhA (T0-T3)	0.355	**0.005**
ΔMPO (T2-T3) vs. ΔIL-6 (T2-T3)	0.735	**<0.001**
ΔIL-6 (T2-T3) vs. ΔMFIS TOT (T0-T2)	0.321	**0.012**
ΔIL-6 (T2-T3) vs. ΔMFIS TOT (T2-T3)	0.405	**0.001**
MFIS Physical (T3) vs. IL-6 (T3)	0.528	<0.001
MFIS Cognitive (T3) vs. IL-6 (T3)	0.456	**<0.001**
IL-6 (T2) vs. MPO (T2)	0.374	0.003
IL-6 (T3) vs. MPO (T3)	0.888	**<0.001**

**Table 7:** Spearman’s rank correlation analysis between clinical and biological variables in CG.

	Spearman’s ρ	*p* Value
IL-6 (T3) vs. MFIS Physical (T3)	0.217	0.096
IL-6 (T3) vs. MFIS Cognitive (T3)	0.206	0.114
IL-6 (T2) vs. MPO (T2)	0.419	0.001
IL-6 (T3) vs. MPO (T3)	0.697	**<0.001**
ΔIL-6 (T2-T3) vs. ΔMFIS TOT (T2-T3)	0.177	0.176
ΔMPO (T2-T3) vs. ΔIL-6 (T2-T3)	0.776	**<0.001**

## Data Availability

The data presented in this study are available on request from the corresponding author due to privacy and ethical restrictions.

## Supplementary Materials

The following supporting information can be downloaded at: https://www.mdpi.com/article/10.3390/nu18213506/s1, Figure S1: CONSORT 2025 Flow Diagram; Table S1: CONSORT 2025 Checklist of information included when reporting this randomised trial [74].
